# Don't Fall Off the Adaptation Cliff: When Asymmetrical Fitness Selects for Suboptimal Traits

**DOI:** 10.1371/journal.pone.0034889

**Published:** 2012-04-11

**Authors:** Elodie Vercken, Maren Wellenreuther, Erik I. Svensson, Benjamin Mauroy

**Affiliations:** 1 Institut Sophia Agrobiotech, UMR 1355 ISA, Institut National de la Recherche Agronomique, Sophia-Antipolis, France; 2 Department of Biology, Lund University, Lund, Sweden; 3 Laboratoire J.A. Dieudonné, UMR CNRS 7351, Université de Nice-Sophia Antipolis, Nice, France; Centro de Investigación y de Estudios Avanzados, Mexico

## Abstract

The cliff-edge hypothesis introduces the counterintuitive idea that the trait value associated with the maximum of an asymmetrical fitness function is not necessarily the value that is selected for if the trait shows variability in its phenotypic expression. We develop a model of population dynamics to show that, in such a system, the evolutionary stable strategy depends on both the shape of the fitness function around its maximum and the amount of phenotypic variance. The model provides quantitative predictions of the expected trait value distribution and provides an alternative quantity that should be maximized (“genotype fitness”) instead of the classical fitness function (“phenotype fitness”). We test the model's predictions on three examples: (1) litter size in guinea pigs, (2) sexual selection in damselflies, and (3) the geometry of the human lung. In all three cases, the model's predictions give a closer match to empirical data than traditional optimization theory models. Our model can be extended to most ecological situations, and the evolutionary conditions for its application are expected to be common in nature.

## Introduction

Evolutionary theory predicts that trait means in populations should evolve towards the value that maximizes fitness [1], which is also a central assumption in most optimality analyses [2]. However, in many cases the evolution of fitness-related traits might be constrained by genetic or physiological trade-offs that cause negative genetic correlations between traits [3]. Fitness in these situations will be maximized in a way that depends on the balance between the counteracting selective forces on traits, and the net fitness functions are expected to be bell-shaped [4]. For instance, increased annual reproductive effort is expected to negatively affect adult survival in long-lived species, leading to a fitness optimum where lifetime reproductive success will be maximized by intermediate reproductive effort [5]. Optimality theory predicts that natural selection will drive the population towards this ‘optimal’ trait value that maximizes fitness [6], which has a close connection to the concept of ‘adaptive peaks’ in population genetics [7]. This classical optimization approach has been successfully applied in evolutionary ecology to predict the population mean of many phenotypic traits.

In particular, the evolution of reproductive traits, such as offspring number, has received considerable interest and has provided evolutionary ecologists with a solid conceptual foundation for optimality theory in life-history evolution [8]–[10]. However, in many species of birds and mammals, the number of offspring most commonly observed is often less than the maximum [11]–[16]. Several alternative theories have been advanced to explain this pattern [15], [17]: (1) costs of reproduction due to trade-offs with parental survival or future reproduction [18]; (2) inter-annual variation in juvenile survival related to variation in environmental quality [12]; (3) individual optimization in relation to individual condition and local resource availability [19]; and (4) the interaction between asymmetrical fitness costs and individual variance in brood size (‘cliff-edge hypothesis’) [12], [20].

Among these hypotheses concerning the evolution of litter size, cliff-edge effects have the potential to provide a unifying framework for understanding the optimization of phenotypic traits. This theory predicts that when juvenile survival is asymmetrically low in large broods, moderate variance around the optimal brood size will result in large differences in survival between clutches slightly smaller or larger than the optimal. As a consequence of these asymmetric costs, females producing larger than the most productive broods will leave fewer descendants than females producing smaller than the most productive broods, and the evolutionary optimal should be smaller than the most productive brood size.

**Figure 1:**
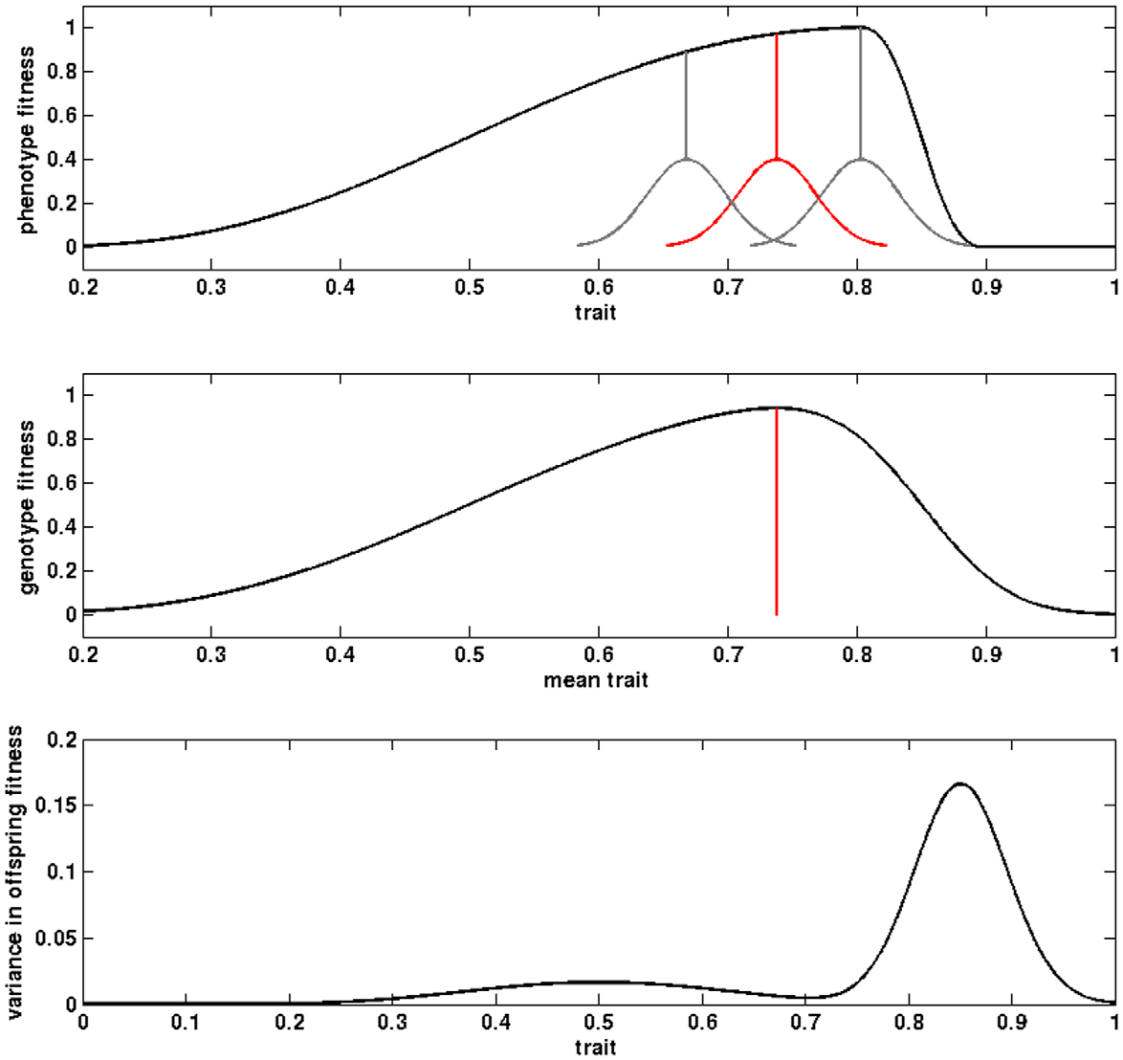
Comparison between (a) the phenotype fitness function, and (b) genotype fitness function relative to the trait variation (after Martin and Huey [**27**]). Because of the steep part (cliff) of function (a), the optimal trait value (red phenotypic distribution) is shifted downwards from the trait value that maximizes phenotype fitness. Function (c) represents the variance in offspring phenotype fitness for different mean values of the trait.

In what follows, the relationship between phenotypic value and reproductive value will be referred to as ‘phenotype fitness’ (which defines the single most productive brood size). In contrast, the relationship between genotypic value and the reproductive value averaged over the phenotype range for each genotype will be referred to as ‘genotype fitness’ (which defines the evolutionary optimum). The difference between these two definitions of fitness is illustrated on Figure 1. In the absence of any phenotypic variance, these two definitions merge.

On a more general note, cliff-edge effects are related to the properties of convex functions known as Jensen's inequality. Jensen's inequality states that, for a convex function *f* and a set of values *x = (x_1_,…,x_n_)* with a mean of 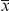 and non-zero variance, the average image of *x*, 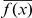 does not equal the image of the average *x*, 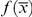. In other words, the trait value that gives the highest genotype fitness in the presence of phenotypic variance is not the value that gives the highest phenotype fitness. These analytical principles have been successfully applied to a wide range of evolutionary questions, including the evolution of reproductive systems and life-history strategies, individual behaviour or population dynamics in variable environments [21]–[28]. In this article, we propose a general mathematical formalization of the cliff-edge problem that can be applied to any fitness-related trait that exhibits phenotypic variability and has asymmetric fitness costs. These conditions are expected to be quite common in nature; hence the generality of a model incorporating these effects is likely to be high.

We develop an analytical model that describes the evolution of a population with random variation in the expression of a fitness-related trait and an asymmetrical fitness function. We demonstrate that these conditions select for apparent sub-optimal genotypes with regard to phenotype fitness, and we show that the optimal genotypic value depends on both the amount of variance of the trait and on the skewness of the fitness function. The model provides quantitative predictions of the position of the optimum and the distribution of phenotypic variance. To illustrate our method, we apply the model to three different evolutionary systems for which we were able to estimate realistic fitness functions from empirical data. Two of these examples (evolution of litter size in guinea pigs, and the evolution of male sexual ornaments in a damselfly) are in line with the classical framework of life history evolution. The third example (evolution of human respiratory tract geometry) stems from evolutionary medicine and demonstrates that cliff-edge effects can act on any trait that is targeted by natural selection.

## Results

### 1- Model of the evolution of a population in presence of phenotypic variation

We consider a population with a continuous trait (e.g. reproductive effort, physiological parameters). We assume that there is no genetic variation for this trait in the population: all genotypes take the value *g*, but the phenotypic expression can vary randomly from *ϕ_min_* to *ϕ_max_*. This phenotypic variance can arise from several different processes (e.g. developmental instability, environmental variability, maternal effects, epistasis) as long as it is a random process such that any individual of genotype *g* can experience any phenotype between *ϕ_min_* and *ϕ_max_*. The simple evolutionary processes involved in the population model were chosen to isolate the effect of phenotypic variability on trait evolution and to increase the generality of model predictions.

The function *a(ϕ,t)* denotes the frequency of individuals in the population having the phenotype value *ϕ* at time *t*. We assume that instantaneous fitness depends on the phenotype, so these individuals have a reproductive rate *b(ϕ)≥0* and a mortality rate of *m(ϕ)>0*. Therefore, at each instant *t* and per unit of time, *m(ϕ)a(ϕ,t)* individuals of phenotype *ϕ* die, while *b(ϕ)a(ϕ,t)* descendants are produced by individuals of phenotype *ϕ*.

For any individual with genotype *g*, the phenotype *ϕ* of its offspring is randomly distributed around the value *g* following the distribution function *G(ϕ,g,σ)*. We consider that *G(ϕ,g,σ)* is a Gaussian function (default hypothesis for a quantitative characters, see [29]) centred around *g* with a variance *σ^2^*.

Therefore, offspring of phenotype *ϕ* are produced by parents of genotype *g* in the proportion *G(ϕ,g,σ)*, regardless of the parents' own phenotype, i.e. there are no cross-generational effects. Therefore, at each time a total quantity 
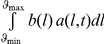
 of new individuals are produced in the population, among which only a fraction *G(ϕ,g,σ)* will have the phenotype *ϕ*.

The variation of the distribution of individuals of parameter *ϕ* over time is then given by the differential equation:
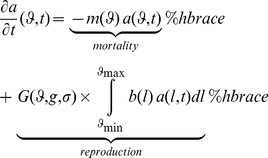
(1)


We show in Supplementary Text S1 that such a population does not go extinct as long as
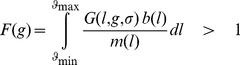
(2)


The term in the integral represents the per capita growth rate *w(l) = b(l)/m(l)* (phenotype fitness) of individuals with phenotype *l* multiplied by their frequency in the population. Thus, the function *F(g)* represents the sum of phenotype fitness of all phenotypes weighted by their respective frequency, i.e. the weighted mean of phenotype fitness in the population. The growth rate function *F(g)* is therefore the genotype fitness of genotype *g*. This result holds for populations with limited resources (Supplementary Text S1).

Then, we consider two populations with limited growth, one with a genotype *g_1_* and the other with a genotype *g_2_≠g_1_*. They are represented by their respective distribution *a_1_(ϕ,t)* and *a_2_(ϕ,t)*. They are interacting with each other due to mutually shared resources that are limited. In order to have true competition, we assume that each population does not go extinct if it is alone, which is equivalent to *F(g_1_)>1* and *F(g_2_)>1*.

The equations that describe the evolution of these populations and their distributions along time are:
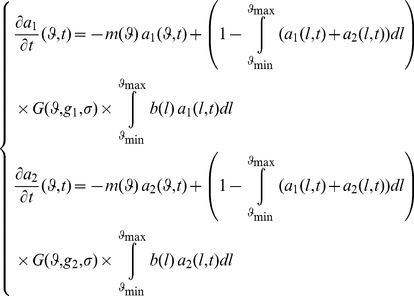
(4)There are four equilibrium points: coexistence of both populations; extinction of both populations; only one population survives while the other goes extinct (two combinations). Coexistence is possible only if *g_1_ = g_2_*, which is excluded by hypothesis. Moreover since *F(g_1_)* and *F(g_2_)* are assumed to be strictly greater than *1* for each population, it can be shown that the extinction of both populations is not possible. Thus, under these hypotheses one population must invade the other. Then, a successful invasion of population *g_1_* into population *g_2_* (i.e. equilibrium *a_1_≠0* and *a_2_ = 0* stable) is possible if and only if:
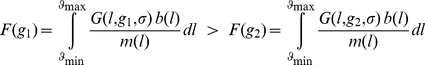
(5)


Hence, the evolutionary stable strategy (ESS) corresponds to the genotype g* that maximises the growth rate function *F(g)*. The population of parameter g*, also called the super-mutant population, will invade any population of parameter g≠g*, while it cannot be invaded by other populations with a parameter g≠g*. Thus, the genotype g* is an ESS and should be observed in population at the equilibrium state, although the phenotype varies in the population.

To determine g*, it is necessary to calculate the maximum of the growth rate function *F(g)*. Hence, the most efficient genotype is the one that maximises the success of the whole population by cumulating the relative success of each phenotypic trait weighted by their frequency.

When the fitness function is symmetric or if there is no phenotypic variance at all, the genotypic value g* associated with the maximum of the function *F* is equal to the value that maximizes the phenotype fitness *w*. However, when the fitness function is asymmetric and the phenotypic variance is non-zero, these two values do not match anymore, as predicted by Jensen's inequality. In this case, the optimum genotype g* value is systematically shifted from the maximum of the phenotype fitness in the direction of the least slope (Supplementary Text S2).

### 2- Application of the model using three biological examples

Most heritable traits are expected to include some degree of non-additive genetic, environmental or developmental variability that affects their optimal expression. Our first example focuses on optimal litter size in laboratory strains of Guinea pigs (*Cavia porcellus*). For this, we re-analyzed Mountford's original data set [20] that was used in his development of the cliff-edge hypothesis. Our second example deals with a secondary sexual trait in the damselfly *Calopteryx splendens*, using survival and mate choice data obtained in the field ([30], M. Wellenreuther, E. Vercken and E. Svensson, unpublished data). The third example is based on modelling work about the impact of lung geometry on respiratory performance in humans [31]. In all these examples, fitness functions are not symmetrically shaped around their maximum value, and we show consistent matching between empirical data and model predictions.

#### 2-1 Example 1: Optimal litter size in Guinea pigs

In his seminal paper, Mountford [20] showed that the litter size that leads to the maximum number of surviving offspring in Guinea pigs was not the most frequent one. He suggested that the phenotypic variability associated with high asymmetric fitness costs for large litter sizes could explain this observation. To prove the validity of his theory, he produced a theoretical example based on simple numeric calculations. In what follows, we show that the frequency distribution of litter size in Guinea pigs could be predicted by the general model described above, and that this model can also provide information about both the optimal genotype as well as the variance in the expressed phenotype.

**Figure 2:**
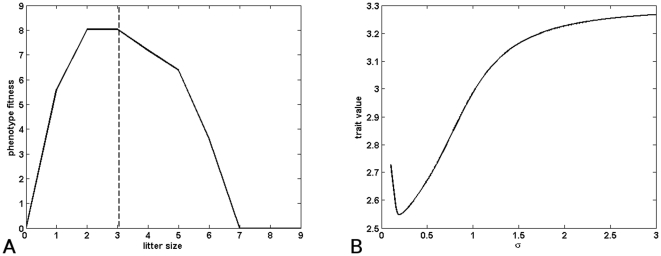
Effect of phenotypic variance on optimal litter size in Guinea pigs. (a) Asymmetric fitness function *b(L)/m(L)*. The curve reaches its maximum value on the plateau between *L = 2* and *L = 3*. The dashed line corresponds to the optimal genotype *L = 3.05* (*σ = 1.14*) that best fits the empirical data from Mountford [20]. (b) Position of the optimal genotype relative to the standard deviation *σ*.

In our model, the litter size is called *L* and extents from *L_min_ = 0* to *L_max_ = 9*. Reproductive rate *b(L)* corresponds to offspring survival in relation to the litter size (data reproduced from Mountford [20]). Life-history theory predicts that, as a consequence of trade-offs between present and future reproduction, female survival should decrease when litter size increases ([32]–[36] but see [15], [37], [38] for counter-examples). In the absence of any data on the precise relationship between litter size and female mortality rate in Guinea pigs, we assumed a simple linear model *m(L) = m_r_+α×L*, where *m_r_* is the mortality rate for non-reproductive individuals and *α* is a constant estimated by least-squares method. The intercept value *m_r_* does not affect the position of the genotype fitness maximum as it plays the role of a scaling factor once *α* is chosen. The phenotype fitness function is asymmetric around a maximum plateau for litter sizes between 2 and 3 (Figure 2a).

The general method to compare the predictions of our model to the measured data is similar in the three different examples used in this paper and is described in detail in Supplementary Text S3. First, we determine the optimal genotype *L_o_(σ)* for each of the possible values of phenotypic variance *σ^2^*. Second, we find the value of (*σ, L_o_(σ)*) that best fits empirical data using the mean-square method, here in the first example, the distribution of litter size in Guinea pigs.

Figure 2b represents the relationship between the standard deviation *σ* and optimal genotype *L_o_(σ)* (first step). Because the phenotype fitness function has a maximum plateau, this relationship is not monotonic. For small values of *σ*, most offspring will fall within the phenotype fitness plateau. However, because of the long tails of the Gaussian distribution, a small proportion will be outside of it. Therefore, the optimal genotype fitness is initially shifted away from the steepest slope (left side).

When *σ* increases, at first a significant proportion of offspring phenotypes reaches values of *L*>3, while fewer phenotypes reach values of *L*<2 (because the optimum is initially right-shifted). Therefore, the optimum genotype value decreases as it shifts away from the closest fitness slope, accounting for the initial negative relationship in Figure 2b.

For higher values of *σ*, a more significant proportion of offspring phenotype reaches values of *L*<2 and thus ‘fall off the cliff’. Because the phenotype fitness loss is higher for phenotypes that reach values of *L*<2 than for those with values of *L*>3, the optimum genotype will shift away from the steepest slope and tends to obtain higher values of *L* as *σ* increases (positive relationship in Figure 2b).

**Figure 3:**
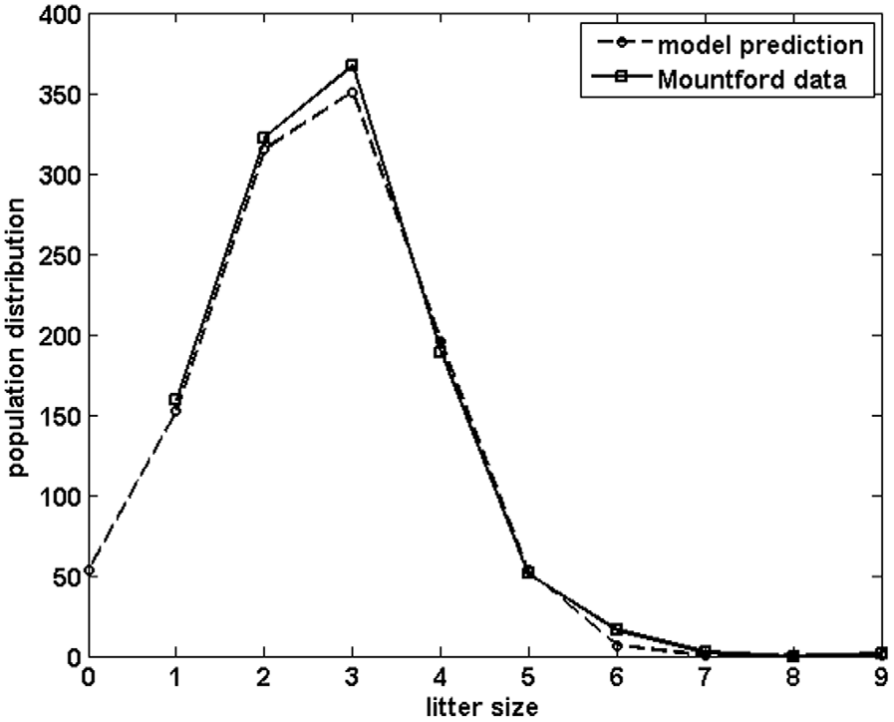
Comparison between model predictions (dashed line) and empirical data (Mountford [**20**], solid line) for the distribution of litter sizes in the population. The model predicts an optimal genotypic value for litter size at *3.05* with a variance *σ^2^* of *1.30*.

In Guinea pigs, the optimal phenotypic trait predicted was *3.05* with a phenotypic variance of *1.30* (*σ = 1.14*). The difference between the distribution of the trait in the population predicted by the model and the distribution observed by Mountford [20] was less than *3.5%* (Figure 3).

#### 2-2 Example 2: Selection on male wing patch size in *Calopteryx splendens*

In the damselfly *Calopteryx splendens*, males have a dark melanized wing patch that covers approximately 50% of the wing [39]. Wing patches in this species function as secondary sexual traits and are only carried by males [40]–[42]. Wing melanization affects male predation risk, as males with larger wing patches suffer higher mortality from avian predators [30]. This trait is also under sexual selection in this population, where female mating response increases with male wing patch size (Supplementary Text S4).

**Figure 4:**
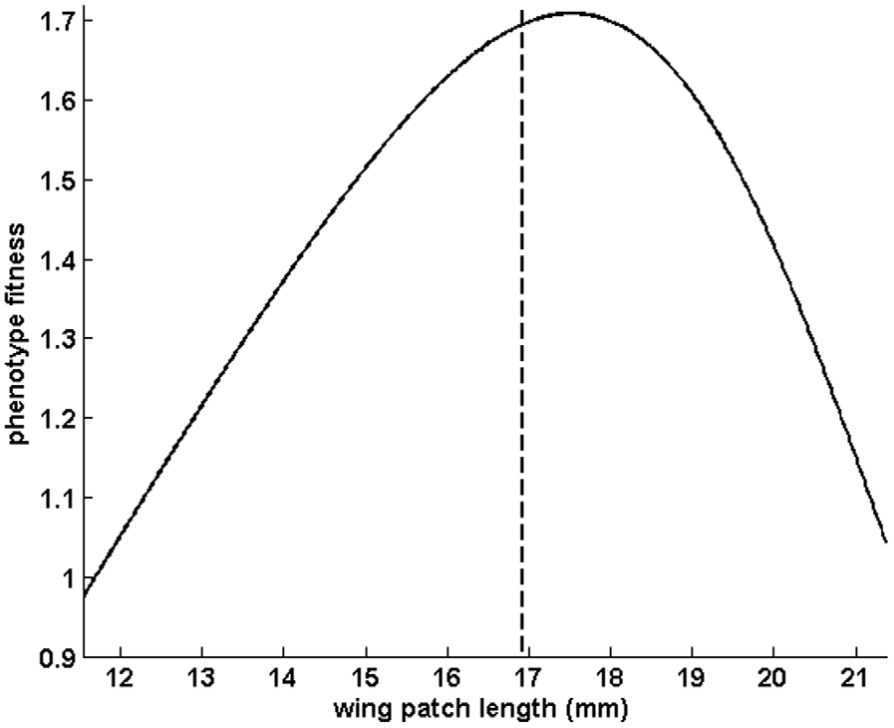
Estimate of the asymmetric phenotype fitness function *z* for *Calopteryx splendens* in relation with wing patch length *x*. The maximum is reached for a wing patch length of *17.52 mm*. The dashed line represents the optimal genotype predicted by our model that best fits the observed population distribution, i.e. a patch length of *16.93 mm*.

The phenotype fitness function is asymmetric (Figure 4) around a maximum at wing patch length *x = 17.52 mm*. The fitness decrease is steeper for larger patches; hence the optimal genotype is expected to be smaller than the phenotype fitness maximum.

**Figure 5:**
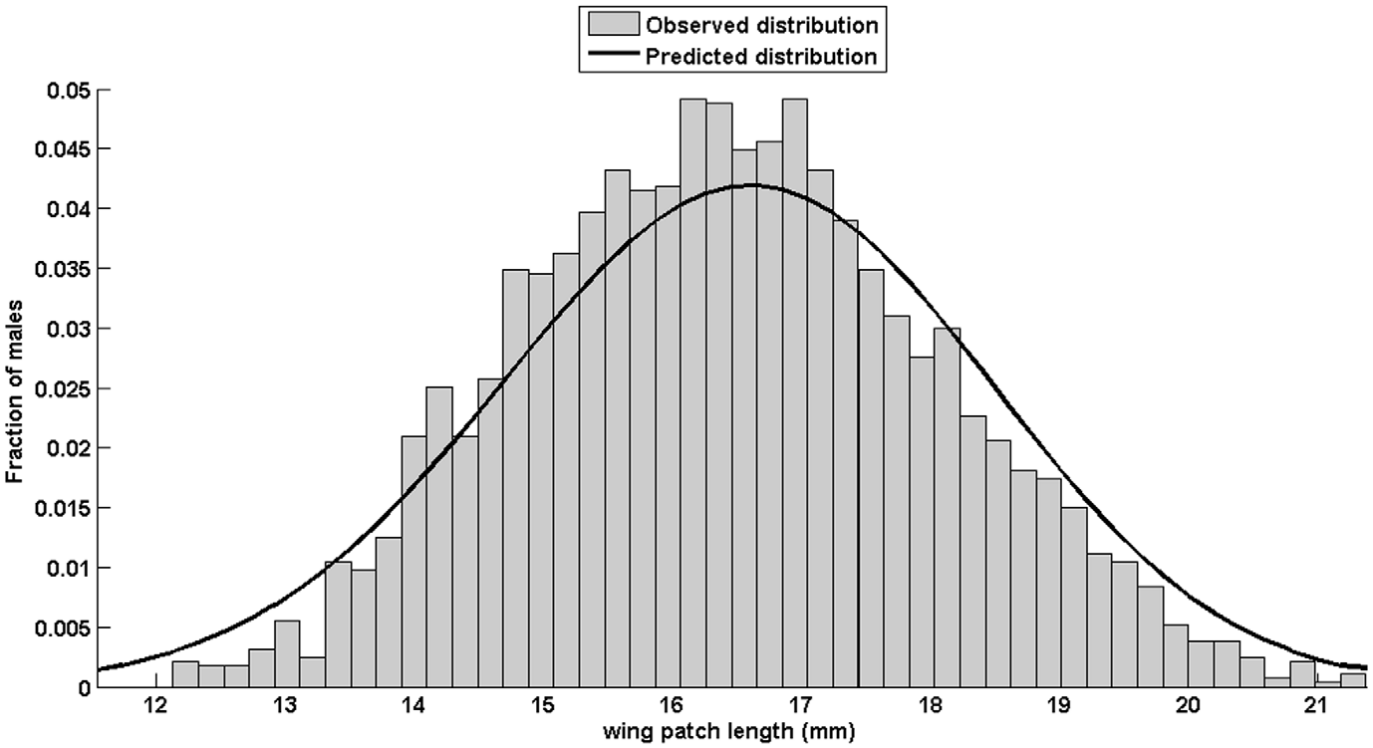
Distribution of wing patch length of *Calopteryx splendens* at the population Naturreservat Klingavälsån (55.6384, 13.54142) in southern Sweden. The bars correspond to males caught in the field. The line shows the distribution predicted by the model with an optimal patch length of *16.93* mm and a standard deviation of 2 mm.

The best fit was obtained with an optimal trait *x_o_* of *16.93 mm* and a standard deviation *σ* of *2 mm*. The difference between the values predicted by the model and those measured in the field was less than *17%* (Figure 5).

#### 2-3 Example 3: Estimating optimal lung geometry

**Figure 6:**
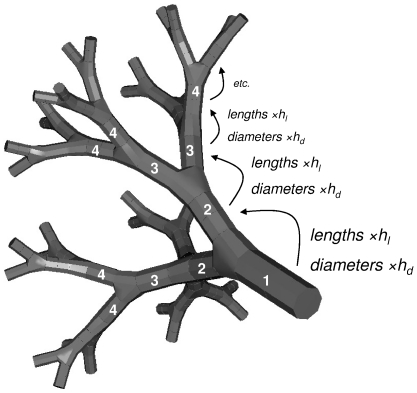
Model of the distal part of the bronchial tree used in this study. After each bifurcation the generation index is incremented by one (white numbers). The full model consists of *11* generations.

Mauroy et al. [31] developed a model of the human bronchial tree to study the relationship between the geometry of the tree and its hydrodynamical resistance. They modelled the distal part of the lungs as a dichotomical tree branching in a homothetical way: at each bifurcation: each branch divides in two identical smaller branches, whose length and diameter are reduced by a constant factor *h*, the homothetical factor (Figure 6, for *h_l_ = h_d_ = h*).

They showed that the mean phenotypic parameter *h*, observed from empirical data [43], is around *0.8470*, while the optimum value predicted by their model was *0.7937*. Although small, this difference is expected to have major effects on the resistance and volume of the lung because of the multiplicative nature of the homothetical transformation (i.e. if the tree bifurcates 10 times, the deepest branches will be *h^10^* smaller than the first generation branch). Large lung hydrodynamic resistance (small *h*) requires more energy for lung ventilation, while large lung volume (large *h*) results in a reduced exchange surface (less volume is available for alveoli). We consider that the fitness of an individual is a function of respiratory efficiency and thus depends on the value of the parameter *h*.

**Figure 7:**
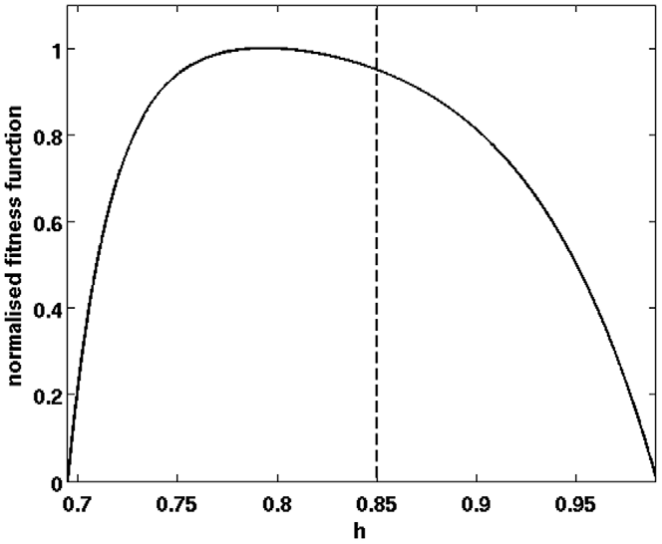
Relationship between homothetical factor *h* and fitness (trade-off between lung volume and hydrodynamical resistance). The vertical dashed line corresponds to the optimal genotype *h = 0.8504* with *σ = 0.2*, which best fits empirical data.

We further extended the model originally formulated by Mauroy et al. [31] to estimate a more realistic fitness function for *h*. Based on morphometric data [44] we assumed that lengths and diameters of the tree branches are not reduced by the same factor at each bifurcation (Figure 6, *h_l_≠h_d_*). Under this hypothesis, the resulting phenotype fitness function (Figure 7) is asymmetrical around the optimum (Supplementary Text S4). The steepest decrease in fitness occurs for values below the optimum, thus we expect the optimal value of the genotype fitness to be higher than the most efficient phenotype.

Empirical data indicate a mean phenotypic value of *h = 0.8470* [36]. The model predicts that this mean phenotype is reached for an optimal genotype *h = 0.8504* and a standard deviation *σ = 0.2*. Compared with the value maximizing the phenotype fitness function *h_min_∼0.7937*, the optimal genotype corresponds to a resistance that is *3.3* times smaller and a volume that is *2.4* times larger. This result quantitatively confirms the hypothesis stated by Mauroy et al. [31] that this shift from the phenotypic optimum acts as a security margin to protect the bronchial tree from phenotypic variations. This analysis is representative of an optimality problem related to the geometry of a transport tree, and as such it can be extended to many other well-known theoretical contexts, such as the Metabolic Theory of Ecology [44], [45].

## Discussion

Environmental variation is ubiquitous in nature and can generate substantial levels of phenotypic variation in fitness-related traits [20], [23], [46]. Such unpredictable variation (e. g. developmental plasticity) can have profound effects on optimal trait values [22], [47]. Our model explicitly incorporates such unpredictable variation through its effects on phenotypic variance and shows that the position of the genotype fitness optimum will ultimately depend on both the amount of phenotypic variance and the shape of the fitness function. For symmetrical fitness and variance functions, the optimal value for the fitness-related trait matches the value that maximizes phenotype fitness. However, this classical optimization scenario does not hold when fitness functions are asymmetrical and when environmental variance leads to a variable expression of genetic traits. Then, the genotype fitness optimum is instead expected to shift from the phenotype optimum value in the direction of the least slope. Such qualitative predictions can be driven directly from the shape of the fitness function. Furthermore, as illustrated in our three examples, quantitative predictions of random phenotypic variance and genotype fitness optima can be derived from empirical data.

### Model applications

In the first two examples, we used empirical data on the frequency distribution of traits and their relationship with fitness components (reproductive rate and mortality) to estimate *σ*, the amount of random phenotypic variance in the population, and to validate the predictions from the model. If the theoretical distribution predicted by the value of *σ* closely matches the observed distribution of the trait, then the cliff-edge hypothesis is a sufficient condition to explain the shift in the distribution away from the phenotype fitness maximum. In this case, the most frequent phenotype in the population is located at the predicted value of the genotype fitness optimum. Furthermore, independent of the intrinsic quality of the prediction, the difference between the theoretical and observed descriptors provides information about the importance of processes other than cliff-edge effects in the evolution of the trait. In these two examples, we obtained less than 3.5% deviation for the Guinea pig data and less than 17% for the damselfly data. Data on litter size in Guinea pigs are more likely to meet the model's assumptions (e.g. laboratory strains with low amount of additive genetic variance, controlled environment with few external selective pressures). In contrast, natural damselfly populations should contain more genetic variability, and many selective pressures in addition to predation and female mate choice are expected to affect the evolution of male wing patch size [40]. These processes are partly responsible for the variability that is not incorporated in the model. Yet, the model gives a closer prediction of the actual fitness optimum than the phenotype fitness maximum, thus supporting the claim that cliff-edge effects are likely to play a strong role in the evolution of natural populations.

Alternatively, the model can be used as an *a priori* hypothesis to predict the value of key parameters when empirical data is not available, which is a classical approach in physics and biomechanics modelling. In the lung example, the frequency distribution of the trait *h* is unknown, but its mean value in the population has been estimated. Mauroy et al. [31] suggested that the value of *h* is shifted from the phenotype fitness optimum in order to confer higher robustness to the lung geometry in response to developmental variation. In this context, the cliff-edge hypothesis provides a formal framework to calculate the expected value of *σ*, which can then be used to implement other models and derive further predictions that can be empirically tested.

### Generality of the conditions of the model

The keystone hypotheses of the model are the existence of an asymmetrical fitness function and a certain amount of phenotypic variance, and the qualitative model predictions appear robust to the precise shape of these functions (Supplementary Text S2). Although the exact geometry of trade-offs will differ between different ecological situations, asymmetrical trade-offs are likely to be the rule rather than the exception, especially for traits under stabilizing selection [48]. Indeed, if a trade-off results from the interaction between two unrelated traits, there is no reason why their respective effects on phenotype fitness should be exactly opposing each other, i.e. completely symmetric. Similarly, there are many processes that can generate random phenotypic variation. For instance, condition-dependence, phenotypic plasticity, developmental effects, environmental fluctuations and/or non-additive genetic effects (e. g. epistasis) are all common and well-known processes that are likely to increase phenotypic variance [49], [50]. These processes are expected to have a significant influence on traits with low heritability, which is a common characteristic of traits strongly related to fitness [29]. Therefore, sources of stochastic phenotypic variation have been suggested to be key factors in the evolutionary ecology of populations [51]–[54].

Finally, we modelled the effects of phenotypic variance during development of individual phenotypes. These phenotypes are then assumed to be stable during life, i.e. we explicitly considered inter-generational phenotypic variance. However, the model can be generalized and the same predictions can also be made in the case of intra-individual variance, for example, when the value of a fitness-related trait changes during an individual's life. Such an example was recently documented by Martin and Huey [27] in the context of thermoregulation in reptiles. The authors showed that the optimal range of body temperatures for an individual should not be centred at the temperature for which the instantaneous fitness is maximised, but should be shifted towards a lower temperature and that the magnitude of the shift increased with the asymmetry of the fitness function.

### Phenotypic variability and species adaptation

In the context of optimization problems, stochastic effects can influence the predictions of theoretical models (e.g. bet-hedging strategies, optimization of the geometric mean fitness, [23], [55], [56], this study). However, these effects depend quite strongly on the relationship between an individual's genotype and the variability of its phenotype.

First, if the variability in phenotype expression is independent from the individual's genotype (as is the case in our model), its association with an asymmetrical fitness functions can be a significant limit to adaptation. Our example of bronchial tree geometry illustrates this situation and provides an adaptive explanation relating to the fact that the human lung is probably not as efficient as it could be. Several other examples in evolutionary medicine appear to be consistent with the existence of cliff-edge effects [57]–[60]. Strong directional selection for traits that are globally advantageous would sometimes drive their mean too close to the ‘fitness cliff’, which could set the stage for counter-selection of extreme phenotypes. Such mechanisms would limit the long-term directional evolution of heightened physiological, mental and immune capacities in humans, and the average performance of individuals would be lower than their maximum potential. These examples tentatively suggest that similar processes might have operated in many different species to constrain the evolution of phenotypic traits within a smaller range than their full physiological potential.

Alternatively, when phenotype variability is related to the individual's genotype, the evolutionary consequences are likely to be quite different. In certain conditions, selection can favour the ability of phenotypes to resist random developmental or environmental perturbations, a process known as canalization [61]. Canalization is expected to be favoured in spatially or temporally variable environments, or in environments connected by high levels of gene flow, because it allows the persistence of high genetic variation and evolutionary potential [62]. In contrast, different genotypes might display different reaction norms in response to environmental variations, i.e. the amount of phenotypic variance and the shape of its distribution can differ between individuals. In this case, asymmetrical variance functions can be selected as a way to compensate for an asymmetrical fitness function by avoiding most detrimental phenotypes (Supplementary Text S2). However, such a strategy could be selected only if environmental variations do not affect the fitness function itself, and if they are restricted within a limited range so that extreme phenotypes will be rare.

### Conclusion

In this study, we propose a simple formalization, validated by three empirical examples, of an evolutionary process known as the ‘cliff-edge’ effect. Our predictions stand for any trait (i) associated with an asymmetrical fitness function and (ii) when phenotypic expression is subjected to random variation, which are conditions expected to be common in nature [63], [64]. In this framework, future studies should aim at analysing the optimization of genotype fitness instead of phenotype fitness. If only cliff-edge effects are shaping the evolution of the trait, then the most frequent value of the trait should match the genotype fitness optimum, i.e. the genotype fitness optimum is the null hypothesis for trait optimization. On the contrary, if the trait is non-optimal with regard to the genotype fitness, other evolutionary processes should be considered. For instance, unmeasured fitness components might cause undetected trade-offs that constrain the adaptation of the trait.

## Supporting Information

Text S1 **Analysis of the population model.**
(PDF)

Text S2 **Influence of the shapes of the phenotype fitness and variance on growth rate function.**
(PDF)

Text S3 **Methods for model fitting to empirical data.**
(PDF)

Text S4 **Detailed analysis of empirical examples 2 and 3.**
(PDF)
