# Supplementary material for: Don't Fall Off the Adaptation Cliff: When Asymmetrical Fitness Selects for Suboptimal Traits
Source: PLoS One. 2012 Apr 11;7(4):e34889. doi: 10.1371/journal.pone.0034889 (PMC3324540; doi:10.1371/journal.pone.0034889)
Supplement: Text S1 — Analysis of the population model. (PDF) [file pone.0034889.s001.pdf]

Analysis of the population model

Detailed mathematical proofs of the following statements will be published separately in the near future. In particular, those proofs will show that the function $a(\varphi,t)$ remains positive within the biological context, i.e. if it is initially positive and if the initial population is not too large.

1. Condition for persistence of a population with unlimited growth

We multiply equation (1) by $b(\varphi)/m(\varphi)$ and integrate the result:

$$\frac{d}{dt}\int \frac{b(l)a(l,t)}{m(l)}dl = \int b(l)a(l,t)dl \times (F(g)-1)$$

If $F(g) \geq 1$, $\int \frac{b(l)a(l,t)}{m(l)}dl$ is either constant or increasing with t, which implies that $a(\varphi,t)$ cannot tend to zero, i.e. the population never goes extinct.

If $F(g)<1$, $\int \frac{b(l)a(l,t)}{m(l)}dl$ is decreasing with time and positive, therefore it converges towards a positive limit and its derivative tends towards zero. $\int b(l)a(l,t)dl$ tends towards zero, i.e. the population always goes extinct.

2. Condition for persistence of a population with limited growth

Equation (1) describes a population with exponential growth, where birth and death rates do not depend on the population density. In most cases though, population growth is limited by the quantity of resources available in the environment, and growth rates decrease

as population size approaches carrying capacity. This phenomenon can be incorporated into equation (1) as follows:

$$\frac{\partial a}{\partial t}(\varphi,t) = -m(\varphi)\,a(\varphi,t) + \left(1 - \int_{\varphi_{\min}}^{\varphi_{\max}} a(l,t)dl\right) \times G(\varphi,g,\sigma) \times \int_{\varphi_{\min}}^{\varphi_{\max}} b(l)\,a(l,t)dl \qquad (3)$$

The term *(1-∫a(l,t)dl)* represents competition for limited resources and implies that if the total population reaches 1 then no more births are possible and the population can grow no further. This is equivalent to a logistic growth term for the total population for a normalized carrying capacity equal to 1.

We can then formulate a semi-explicit solution of equation (3) of the form:

$$a(\varphi,t) = a_0(\varphi)e^{-m(\varphi)t} + G(\varphi,g,\sigma)\int_0^t \lambda(s)e^{m(\varphi)(s-t)}ds$$

and $\lambda(t) = (1 - \int a(l,t)dl)\int b(l)a(l,t)dl$

Two limits, 0 and $(1-1/F(g))(\int G(l,g,\sigma)/m(l)dl)^{-1}$, are possible for λ when t goes to infinity. It can be shown that if we consider only positive solutions of eq. (1) then $F(g) \leq 1$ is equivalent to $\lambda(t) \rightarrow 0$ when $t \rightarrow \infty$, i.e. the population goes extinct. Alternatively, if $F(g) > 1$ then $\lambda(t) \rightarrow (1-1/F(g))(\int G(l,g,\sigma)/m(l)dl)^{-1}$ strictly positive, i.e. the population persists.

In this case, the asymptotic distribution of phenotypes in the population is given by:

$$a_s(\varphi) = \frac{G(\varphi,g,\sigma)}{m(h)}\left(\int_{\varphi_{\min}}^{\varphi_{\max}} \frac{G(l,g,\sigma)}{m(l)}dl\right)^{-1} P$$

where $P = \int_{\varphi_{\min}}^{\varphi_{\max}} a(l)dl = 1 - 1/\left(\int_{\varphi_{\min}}^{\varphi_{\max}} \frac{G(l,g,\sigma)b(l)}{m(l)}dl\right)$

This relationship was used to calculate the theoretical distributions expected under the cliff-edge hypothesis in the three empirical examples we developed.
