# Supplementary material for: Don't Fall Off the Adaptation Cliff: When Asymmetrical Fitness Selects for Suboptimal Traits
Source: PLoS One. 2012 Apr 11;7(4):e34889. doi: 10.1371/journal.pone.0034889 (PMC3324540; doi:10.1371/journal.pone.0034889)
Supplement: Text S2 — Influence of the shapes of the phenotype fitness and variance on growth rate function. (PDF) [file pone.0034889.s002.pdf]

Influence of the shapes of the phenotype fitness and variance on growth rate function

We recall that the phenotype fitness function for a trait is defined by *w(l)=b(l)/m(l),* and that the phenotype variance function is *G(l,g,σ)*. Then, the growth rate function, which maximum is the optimal genotype, is

$$F(g) = \int_{\varphi_{\min}}^{\varphi_{\max}} G(l,g,\sigma)w(l)dl\,.$$

The growth rate function shape and the optimal genotype are thus dependent on the shapes of both the phenotype fitness function and the phenotype variance function. We explored the respective influence of the presence and direction of asymmetry in one or the other of these functions on the shape of the growth rate function and the position of the optimal genotype.

The results of this analysis are shown on Figure 8. When neither the phenotype fitness nor the phenotype variance is asymmetrical, the optimal genotype coincides with the optimal phenotype (A). When the phenotype fitness function only is asymmetrical, the maximum of the growth rate function is shifted away from the cliff (B) towards the softest slope of the phenotype fitness function (main case discussed in the text). If the phenotype variance function only is asymmetrical, then the shift is in the direction of the cliff of the phenotype variance (C and E). Therefore, cliff-edge effects are possible even when the fitness function is symmetrical, provided that the variance function is asymmetrical.

When both phenotype functions are asymmetrical and have their cliffs on the same side, then both cliffs compensate each other, and the optimal genotype is less shifted or shifted in the opposite direction (D) than for symmetrical phenotype variance. Finally, when the phenotype fitness function and phenotype variance function asymmetries are on opposite

sides, then their effects add each other and the shift of the optimal genotype is amplified (F) in comparison with the symmetrical phenotype variance.

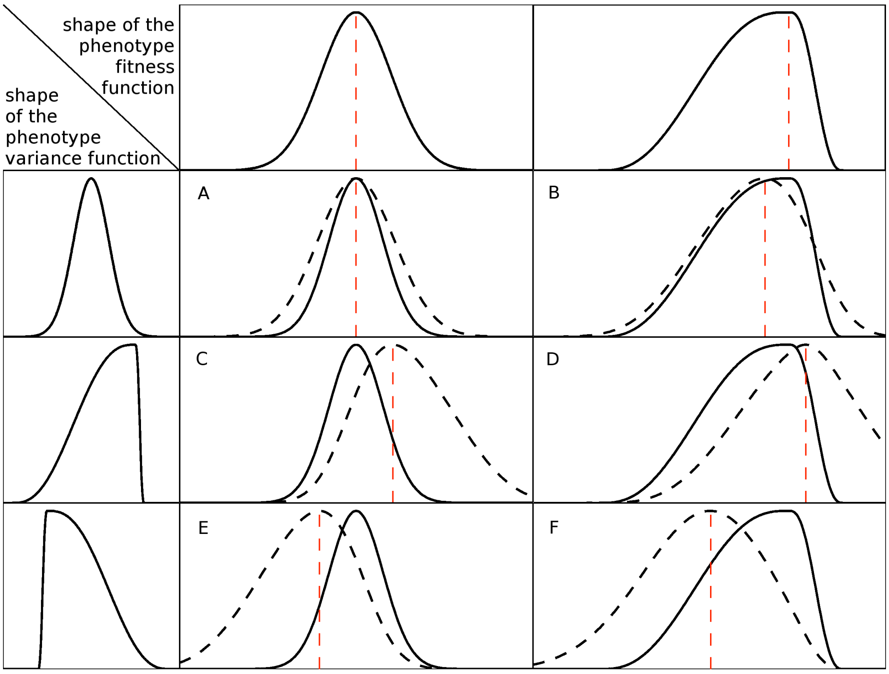

Figure 8: Shapes (black dashed curves) and optimal genotypes (red dashed lines) of growth rate functions computed for various shapes of the phenotype fitness function (horizontal) and phenotype variance function (vertical).
