# Supplementary material for: Don't Fall Off the Adaptation Cliff: When Asymmetrical Fitness Selects for Suboptimal Traits
Source: PLoS One. 2012 Apr 11;7(4):e34889. doi: 10.1371/journal.pone.0034889 (PMC3324540; doi:10.1371/journal.pone.0034889)
Supplement: Text S3 — Methods for model fitting to empirical data. (PDF) [file pone.0034889.s003.pdf]

Methods for model fitting to empirical data

For simplicity, we describe the method using the Guinea pig example. The first step is to determine the optimal genotype given the possible values of the variance of the phenotype σ^2. Thus, for a range of σ chosen to be compatible with the physiology of the species, we estimate the trait $L_o(\sigma)$ that maximises the function

$$F(L) = \int_{L_{\min}}^{L_{\max}} \frac{G(l, L, \sigma)\, b(l)}{m(l)}\, dl$$

This will determine the optimal genotype, given the variance σ^2 in the phenotypes. In the case of the Guinea pig example, the chosen range for σ was *[0, 3]*.

The second step is to determine the most plausible genotype given the measured data, which is determined based on the population distribution in relation to the litter size. For each combination of $(L_o(\sigma),\sigma)$, the proportion of individuals having the phenotype L is given by (see Supplementary Text S1)

$$a(L) = \frac{G(L, L_o(\sigma), \sigma)}{m(L)} \left(\int_{L_{\min}}^{L_{\max}} \frac{G(l, L_o(\sigma), \sigma)}{m(l)}\, dl \right)^{-1}$$

Then, we perform a mean-square method to estimate which combination of $(L_o(\sigma),\sigma)$ gives the best fit to the experimentally measured distribution. Finally, we calculate the relative quadratic error between the observed and the predicted distributions to estimate model fitting quality.
