# Supplementary material for: Don't Fall Off the Adaptation Cliff: When Asymmetrical Fitness Selects for Suboptimal Traits
Source: PLoS One. 2012 Apr 11;7(4):e34889. doi: 10.1371/journal.pone.0034889 (PMC3324540; doi:10.1371/journal.pone.0034889)
Supplement: Text S4 — Detailed analysis of empirical examples 2 and 3. (PDF) [file pone.0034889.s004.pdf]

Detailed analysis of empirical examples

1. Natural and sexual selection in the damselfly *C. splendens*

Based on the data from Svensson and Friberg [30] from a population of *C. splendens* in southern Sweden (Naturreservat at the Klingavälsån river), we fitted the relationship between male wing patch length x and survival rate s within a season with a logistic model (statistical support for the relationship p<0.001):

$$s(x) = \frac{e^{11.544-0.561\times x}}{1+e^{11.544-0.561\times x}}$$

In the same population, male mating success was estimated experimentally in the summer of 2007 by the authors with a simple behavioural protocol (developed by Svensson et al. [42]). Males were caught with butterfly nets and then aged to test if they were mature, following the guidelines proposed by Tynkynnen et al. [I,II]. Only mature males with fully hardened wings were used in the study. The males were then tied around the thorax with a cotton thread that was attached to a 1.5 m long bamboo stick. These tethered males were subsequently presented to at least 5 sexually mature *C. splendens* females in the field. Female mating responses towards tethered males was then observed and the females' sexual responses were scored using a nominal scale ranging from 0 (female attacks the male) to 10 (tandem pair formation) (for details about the behavioural scale see [42]). This experimental design allowed us to quantify the courtship responses of females towards males with different wing phenotypes in the field. To increase the range of variation in male wing patch size, wing patches were also artificially increased using dark paint, and female responses towards males were measured before and after the manipulation. We fitted the relationship between wing patch length x and female response r with a linear model (statistical support: p<0.01).

$$r(x) = 0.174\times x - 1.024$$

Reproductive rate $b(x)$ is proportional to individual survival $s(x)$ and mating success (proportional to female response $r(x)$). Therefore $b(x)=\alpha \times s(x) \times r(x)$.

Adult *C. splendens* never survive from one season to the other (average survival time around 2 weeks, [30]), thus there is no overlap between generations (i.e. $m(x)=1$). Note that the survival rate $s(x)$ corresponds to the survival within the season (i.e. until reproduction) and not from one season to the other.

The phenotype fitness function is: $z(x)= \alpha \times r(x) \times s(x)/m(x)$

In accordance with the general method, to predict the optimal genotype $x_o(\sigma)$ for a given variance σ^2, we estimated the patch length that maximises the value of the function:

$$F(x) = \alpha \int_{x_{\min}}^{x_{\max}} G(y,x,\sigma)\, r(y)\, s(y)\, dy$$

Note that because α is assumed to be a constant, the position of the maximum F does not depend on α. To determine the values of $(x_o(\sigma),\sigma)$ that give the best fit, the population distribution given by the model for σ varying in $[0,3.3]$ *(mm)* and the distribution observed in the field were compared using a mean-square method.

2. Respiratory efficiency function

Empirical data show that bronchi lengths and diameters do not vary in the same way along the bronchial tree [43]. Thus at each generation i, we can define a parameter $h_{l,i}$ and a parameter $h_{d,i}$ that control respectively the reduction of lengths and diameters. Under this hypothesis, an optimisation process similar to that of Mauroy et al. [31] could be performed. The hydrodynamic resistance R and the volume V of a tree that branches symmetrically (a mother branch bifurcates into two identical daughter branches) N times (see Figure 6) are:

$$R = R_0\left(1+\sum_{i=1}^{N}\frac{1}{2^i}\frac{h_{l,1}\times\ldots\times h_{l,i}}{(h_{d,1}\times\ldots\times h_{d,i})^4}\right) \text{ and } V = V_0\left(1+\sum_{i=1}^{N}2^i\times(h_{l,1}\times\ldots\times h_{l,i})\times(h_{d,1}\times\ldots\times h_{d,i})^2\right)$$

V_0 and R_0 are the tree root volume and resistance, respectively. Because resistance and volume are linearly dependent on the length and thus on the $h_{l,i}$, the optimisation cannot give any information on the h_l parameters. This observation shows that the optimisation problem has to be carried out relatively to the sole parameters h_d. The parameters h_l are thus fixed and determined according to the model of Weibel [43]. Doing so leads to an optimal tree whose parameters h_d verify $h_{d,i}=(1/2)^{1/3}$ for all $i=2,\ldots,N$, while only $h_{d,1}$ depends on the data.

The fitness function of $h=h_d$ given by this new optimisation process has a non symmetrical behaviour around that optimum (Figure 7). Moreover, it decreases steeply below the optimal value and smoothly above. This is a consequence of the hydrodynamic resistance (resistance to air circulation) varying in $1/h_d^4$ while the volume varies in h_d^2. To determine the optimal genotype g for a given standard deviation σ, we assumed that the respiratory cost $m(h)$ is inversely proportional to the fitness function $L(h)$. The interpretation of this hypothesis is the following: smaller h ($h<(1/2)^{1/3}$) correspond to large lung hydrodynamic resistances and imply the necessity to allocate a large quantity of energy to lung ventilation, whereas larger h ($h>(1/2)^{1/3}$) correspond to bronchial trees with large volume and imply reduced exchange surface (less volume is available for alveoli). In both cases, there is an energetic cost which influences respiratory efficiency. Thus, the energetic cost will be defined by $m(h)=min(1,m_r\times L(h)/L_{max})$ where L_{max} is a normalization constant, fixed to $L_{max}=L((1/2)^{1/3})$ and m_r is the minimal energetic cost reached by individuals which maximizes the fitness function, here we assume $m_r=0.1$.

As outlined previously, the optimal genotype $h=g(\sigma)$ for standard deviations σ in the range $[0, 0.6]$ was computed to find the value of σ that best fits our criterion, which is, in this case, the mean value of the population. We recall that the optimal genotypes are calculated by finding the maximum value of the effective growth function $F(h)$.

ADDITIONAL REFERENCES

[I] Tynkkynen K, Rantala MJ, Suhonen J (2004) Interspecific aggression and character displacement in the damselfly *Calopteryx splendens*. J Evol Biol 17:759-767.

[II] Tynkkynen K, Kotiaho JS, Luojumäki M, Suhonen J (2005) Interspecific aggression causes negative selection on sexual characters. Evolution 59: 1838-1843.
